# Special Issue “Plant Virus Ecology and Biodiversity”

**DOI:** 10.3390/v11080676

**Published:** 2019-07-24

**Authors:** Ulrich Melcher

**Affiliations:** Department of Biochemistry and Molecular Biology, Oklahoma State University, Stillwater, OK 74078-3035, USA; ulrich.melcher@okstate.edu

I thank all the teams of authors, the scientists who reviewed submitted manuscripts and made suggestions that improved the reports, and the editorial staff workers who put this special issue together. Thanks for your contributions.

The articles published in this special issue highlight many of the processes that contribute to the evolution of viral genomes. A major theme is the reassortment of multi-partite genomes. At its extreme, this results in genomes such as that of *Charavirus canadensis*, in which genome segments have origins from viruses related to *hepacivirusees*, *benyviruses*, *tobamoviruses*, and *hepegiviruses*. As the number of canonical genome segments increases, so will the number of viruses with missing genome components that may have to be discovered. A report of the discovery of the missing components of the subterranean clover stunt virus is presented, authenticating its identification as a *Nanovirus*. Bean-associated *cytorhabdovirus* is proposed as a virus found in resistant bean cultivars. 

Ecology has also played a role in virus spread. An investigation of disease in cyclamen revealed the presence of a strain of the fig mosaic virus. A review of chickpea stunt disease underscored the importance of plant–virus–insect interactions. Glasa et al report an analysis of turnip mosaic virus isolates from pepper, which confirmed that recombination played an important role in the evolution of this virus and the shaping of its genome.

Thanks to all.

